# Interventional Tools to Improve Prescription and Adherence to Medical Plans

**DOI:** 10.1155/2015/602078

**Published:** 2015-11-24

**Authors:** Elísio Costa, Anna Giardini, Alexandra Prados-Torres, Caitriona Cahir, Alessandra Marengoni

**Affiliations:** ^1^UCIBIO, Department of Biological Sciences, Faculty of Pharmacy, University of Porto, Rua de Jorge Viterbo Ferreira No. 228, 4050-313 Porto, Portugal; ^2^Psychology Unit of the S. Maugeri Foundation, IRCCS, Scientific Institute of Montescano (PV), Via Per Montescano 31, 27040 Montescano, Italy; ^3^Spanish National Health System, Aragon Health Sciences Institute (IACS) and Department of Preventive Medicine, Zaragoza University, Via Universitas 36, 50017 Zaragoza, Spain; ^4^Department of Pharmacology and Therapeutics, Trinity College Dublin, College Green, Dublin 2, Ireland; ^5^Department of Clinical and Experimental Sciences, University of Brescia, Viale Europa 11, 25123 Brescia, Italy

In developed countries, nonadherence to treatment in patients with chronic diseases ranges from 30% to 50%, and this rate is even higher in developing countries [1, 2]. Indeed, medication adherence and persistence are recognized as a worldwide public health problem and a challenge for researchers and health care providers, since efforts and interventions to improve patient's adherence and persistence appeared to be ineffective [3].

Nonadherence to medical plans is manifests at every level of the population, but particularly in older adults due to the high number of coexisting chronic diseases and geriatric syndromes and the consequent polypharmacy [4, 5]. Polypharmacy is often associated with inappropriate prescriptions, drug-drug and drug-disease interactions, prescription cascade, which can all increase the risk of adverse drug reactions and therefore the discontinuation of treatment [6]. In addition, the management of chronic diseases requires the patient's continuous psychological adaptation and behavioral reorganization to face recurrent changes in therapeutic indications.

In the literature, many interventions to improve medication adherence have been implemented in different clinical conditions. However, most interventions showed low effectiveness not only in improving adherence, but also in other outcomes, namely, quality of life, health outcomes, and health care costs [7].

This special issue proposed in the context of a collaborative work of A1 Action Group on Prescription and Adherence to Medical Plans of the European Innovation Partnership on Active and Healthy Ageing (EIP-AHA) includes two reviews and three original research articles. In one of the review articles, C. Jäger et al. showed that regular receipt of an updated and comprehensive medication list may reduce patients' concerns and increase the perceived necessity of their medication. This paper supports not only the demand to establish standardized, high-quality medication reviewed lists, but also the need to improve the communication between health care providers (physicians, clinical pharmacists, etc.) and patient's communication. In the other review article, W. Y. Lam et al. revised the validated and commonly used medication adherence measures with the general aim to identifying nonadherence in everyday situations. Concerning the three original articles, M. Lelubre et al. described the experience of a well-established adherence program in Lausanne, Switzerland. The intervention comes from an interdisciplinary collaboration between all healthcare professionals and includes motivational interviews, electronic pill monitors, and reports. It is committed to patients affected by chronic conditions experiencing or at risk of experiencing medication adherence problems. Y.-C. Li et al. studied the effects of adherence to statin therapy on health care outcomes and utilizations in Taiwan. The authors showed that good medication adherence brings better outcomes and saves on medical costs for patients who were taking statin medications. Finally, S. S. Allemann et al. analyzed the general prescription patterns of split tablets in Switzerland and its implications for community pharmacies, patients, and patient care organizations. The authors showed that prescription of fragmented tablet is frequent and it represents not only a safety issue for the patient, but also a pharmaceutical care issue for the pharmacist.

We believe that the reasons behind poor adherence in persons affected by chronic diseases and prescribed with polypharmacy are multifactorial and complex, related to social and economic aspects, health systems and professionals characteristics, specific diseases, and individual patient's features. This is possibly the reason why measurements of adherence and interventions to improve it are so challenging. This special issue on adherence to medical plans will contribute to increase and spread of knowledge on already available but also new scientific evidence on this topic. Of course, further research is needed. We would suggest that a comprehensive approach based on a multistep and interdisciplinary strategy would be helpful in planning intervention programs and new strategies able to increase adherence and impact on major clinical outcomes.
